# Foreword

**DOI:** 10.1093/rb/rbw013

**Published:** 2016-03-29

**Authors:** James M. Anderson

**Affiliations:** Case Western Reserve University USA

This special issue of *Regenerative Biomaterials* presents 13 manuscripts provided by participants of the China–US Forum on Grand Challenges for Biomaterials in the 21^st^ Century. This forum was held on November 17, 2015, in Chengdu, China and was chaired by the Editor of *Regenerative Biomaterials*, Professor Xingdong Zhang and Professor Nicholas Peppas. The participants of this forum were internationally recognized experts from the United States of America and the Peoples Republic of China. It is noteworthy that the United States speakers were members of the National Academy of Engineering, Section 2. Bioengineering, and most of the Chinese speakers were members of the Chinese Academy of Engineering or the Chinese Academy of Sciences. The overlying theme of this forum and this resultant special issue is the impact that the biological environment in which biomaterials will be utilized is now recognized as providing a wide variety of significant and impactful challenges that must be overcome if biomaterials are to achieve clinical application.

While significant advances have been made over the past half century regarding biomaterials, it can be stated that they have produced a clearer vision of the significant challenges that lie ahead for scientists, engineers and clinicians working in the biomaterial arena. This special issue, in part, identifies these challenges from a wide and varied perspective. If significant advances are to be made in the development and clinical use of biomaterials in the 21^st^ century, these challenges must be met and a more in-depth understanding of the biological environment and its interaction with biomaterials must be more fully appreciated as well as utilized.

This special issue presents perspectives which have a wide ranging view of the research and development of biomaterials, translational research involving biomaterials, and the production and manufacturing of biomaterials for clinical application. In particular, these manuscripts provide insight and perspective regarding the response of biomaterials to the physiological and biological environments, evaluation and perspectives of biomaterial biocompatibility, biodegradable magnesium alloys, the utilization of microfluidics to address biomanufacturing, the significance of porosity in defining biocompatibility, the translation from replacement medicine to tissue engineering, regeneration strategies for the central nervous system, limb regeneration as a model for regenerative engineering, new approaches to bacteria-repellant materials and anti-bacterial polymers, biomaterial structure/property relationships as a key to medical device development, and challenges in the scale-up, production and manufacturing of new biomaterials for clinical application.

While these respective manuscripts provide an important list of challenges to be met in the 21^st^ century, these manuscripts also identify gaps in knowledge and information that must be addressed in the future design and development of next generation biomaterials, medical devices and prostheses.

**Figure.**
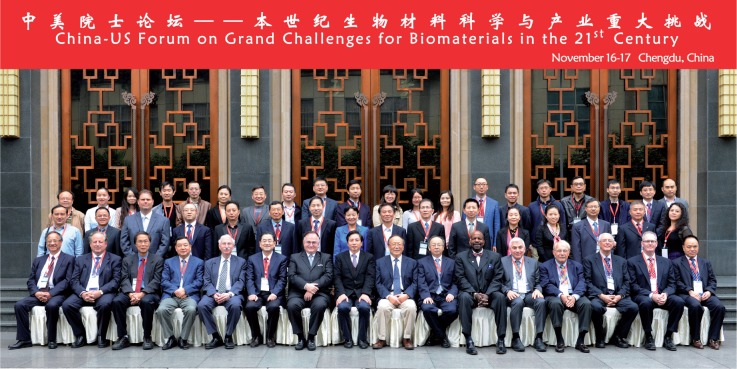
Group Photo for the China–US Forum on Grand Challenges for Biomaterials in the 21st Century

